# The Effectiveness of Palliative Care Interventions in Long-Term Care Facilities: A Systematic Review

**DOI:** 10.3390/jpm14070700

**Published:** 2024-06-28

**Authors:** Xuan Liu, Yun-Chen Chang, Wen-Yu Hu

**Affiliations:** 1 Department of Nursing, Mackay Memorial Hospital, New Taipei City 251020, Taiwan; mmh.f630@mmh.org.tw; 2 School of Nursing, College of Medicine, National Taiwan University, Taipei 106335, Taiwan; 3 School of Nursing, Graduate Institute of Nursing, China Medical University, Taichung 406040, Taiwan; 4 Nursing Department, China Medical University Hospital, Taichung 404327, Taiwan; 5 Department of Nursing, National Taiwan University Hospital, Taipei 100229, Taiwan

**Keywords:** palliative care program, long-term care facility, palliative care

## Abstract

The increasing elderly population is driving higher utilization rates of long-term care facilities, where residents often have multiple chronic diseases, making them potential candidates for palliative care. Timely palliative care interventions can improve their quality of life and medical autonomy. This study systematically reviews the effectiveness of palliative care programs in long-term care facilities. Databases such as PubMed, EMBASE, Cochrane Library, and Airiti Library were searched up to 31 December 2023, using PICO criteria and the following keywords: ‘care home’, ‘nursing home’, ‘residential aged care facility’, and ‘long-term care facility’ for patients; and ‘Gold Standard Framework in Care Homes’, ‘integrated care pathway’, ‘care home project’, and ‘palliative care program’ for interventions. Seven articles were included. The results indicate that the Program of All-Inclusive Care for the Elderly (PACE) intervention did not significantly influence overall quality of life but did improve the quality of death. There were no statistical differences in comfort or quality of death between the dementia and non-dementia groups. However, PACE significantly reduced healthcare costs. The implementation of the Liverpool Care Pathway (LCP) notably enhanced the control of terminal symptoms, while the Gold Standard Framework in Care Homes (GSFCH) effectively improved end-of-life care rates, do-not-resuscitate (DNR) signing rates, advance care planning (ACP) completion rates, and reduced inappropriate readmission rates. While palliative care interventions are shown to improve the quality of end-of-life care, their practical application should be adapted to fit the implementation conditions and capabilities of domestic long-term care facilities.

## 1. Introduction

Taiwan has become an aging society; the elderly population has steadily increased. As of April 2024, the elderly population accounted for 18.65% of the total population, with an aging index of 160.33, compared to 85.7 in 2014, representing an increase of 74.63 [1]. According to long-term care surveys, in 2022, there were approximately 610,000 disabled individuals aged 65 and above, and this number was estimated to increase to 950,000 by 2032. There are around 55,150 long-term care and nursing home staff, with 38,181 staff members in nursing homes and mental healthcare facilities, with an occupancy rate of nearly 85% [2]. The average age of institutional residents is over 80 years old, with each resident suffering from an average of three or more chronic diseases. As diseases progress, health conditions deteriorate, and care needs become more complex, highlighting the importance of early intervention in palliative care. Domestic and international studies have shown that palliative care interventions can reduce ineffective medical treatments for residents and achieve a peaceful end-of-life while reducing medical care costs. Palliative care in Taiwan has been implemented for over 30 years since 1990, extending its service population from terminally ill cancer patients to the top ten non-cancer patients. The service types have also expanded from home, hospital, and shared care to community care. However, the current community-based palliative care mainly focuses on home and community hospital models, rarely reaching the overall residents and staff of long-term care facilities. There is a need for the comprehensive integration of long-term care and palliative care to avoid service discontinuity and provide continuous care for institutional residents.

In the context of long-term care facilities, palliative care (PC) is defined as a specialized medical approach to improve the quality of life for residents suffering from serious illnesses. This approach is centered on relieving the symptoms, pain, and stress of a serious illness—regardless of the diagnosis. The goal is to optimize quality of life by anticipating, preventing, and treating suffering. Palliative care involves a broad spectrum of services, including physical, emotional, social, and spiritual support tailored to the patient’s needs and preferences. In long-term care settings, palliative care is not limited to end-of-life situations but is integrated into the ongoing care plan from the point of diagnosis of any serious, potentially life-limiting illness. This is distinct from hospice care, which is specifically aimed at end-of-life care and typically implemented when the patient is expected to live six months or less. Recent studies in the literature underscore the evolving scope and implementation of palliative care in long-term facilities. For instance, a study highlights integrating palliative care practices to significantly improve symptom management and patient comfort in nursing homes [3]. Another study emphasizes the need for tailored palliative care programs that accommodate the complex needs of long-term care residents, illustrating successful outcomes in patient-centered care and family satisfaction [4].

According to a survey report by the National Health Administration, nearly 80% of middle-aged and elderly individuals have been diagnosed with at least one chronic disease (77.1%), increasing to over 90% (92.4%) for those aged 75 and above. Additionally, more than half (51.3%) report having been diagnosed with three or more chronic diseases. The most common chronic diseases include hypertension, cataracts, diabetes, heart disease, and joint diseases [5]. The combination of multiple chronic diseases and old age has led to discussions on advance care planning, end-of-life quality of life, and preferences for care. One study found that when individuals are in advanced stages of illness or experience severe cognitive difficulties, over 90% prefer not to undergo life-prolonging treatments. Instead, they favor active discussions with healthcare professionals about medical interventions [6]. Most lean toward palliative care to avoid ineffective medical measures and to peacefully transition from life. Suppose staff in long-term care facilities receive sufficient training in palliative care and have adequate resources and manpower. In that case, they can develop the necessary skills to care for residents in their end-of-life stages, reducing the likelihood of inappropriate hospital transfers [7]. Early palliative care interventions in long-term care facilities have been shown to increase the rate of in-facility deaths from 15% to 36.9%. This is achieved through initiatives such as holding palliative care discussions and advocacy meetings, which enhance the awareness of residents and their families regarding end-of-life care [8].

In Taiwan, the implementation of palliative care programs in long-term care settings has been increasingly influenced by guidelines and initiatives from the Ministry of Health and Welfare, reflecting a shift toward integrating globally recognized practices with local healthcare strategies. These programs are designed to identify residents’ palliative care needs, facilitate advanced care planning, and enhance collaboration among healthcare providers. This approach aims to improve symptom management, support the holistic needs of residents with end-stage diseases, and provide care that aligns with family expectations and patient dignity.

While Taiwan has looked to models such as the Gold Standard Framework in Care Homes (GSFCH) and the Liverpool Care Pathway (LCP)—now known as the Integrated Care Pathway (ICP) since July 2013—for foundational concepts, the focus has increasingly shifted toward adapting these frameworks to better suit the local context. This includes training palliative care professionals and institutional staff for periods ranging from 6 to 18 months to support the practical implementation of these models within Taiwanese facilities.

The emphasis is now on creating a tailored system that not only incorporates successful international practices but also addresses the specific needs identified in recent reports by the Ministry of Health and Welfare. These efforts are part of a broader strategy to enhance the quality of life for long-term care residents in Taiwan through improved palliative care services, ensuring that the interventions are both culturally relevant and aligned with national healthcare goals.

In clinical care experiences, it is often observed that staff in nursing homes face increased stress when caring for residents in end-of-life situations, often with limited resources and capabilities. Unfortunately, situations arise where residents have passed away, yet emergency medical systems are still utilized to transport them back to hospitals. Studies have shown that with the intervention of palliative care teams, the rate of end-of-life deaths in nursing homes increased from 57% to 72%, and the number of emergency readmissions decreased. In a study by the Gold Standard Framework (GSF) in 2012, the intervention group had 200 readmissions compared to 550 in the control group. Implementing appropriate palliative care measures in long-term care facilities prevents and avoids ineffective medical care and enhances the quality of care and end-of-life experience for residents. Additionally, it can increase the professional autonomy of healthcare professionals within these facilities. Given the above, this paper aims to conduct a systematic literature review to explore the implementation of palliative care interventions in current long-term care facilities. By utilizing palliative care interventions to improve the quality of end-of-life care, the aim is to synthesize comprehensive and appropriate care strategies, provide recommendations for long-term care facilities regarding palliative care, and offer insights for future research in this area.

## 2. Materials and Methods

### 2.1. Search Strategy

The data collection for this systematic literature review was conducted through searches in the PubMed, EMBASE, Cochrane Library, Airiti Library, and National Digital Library of Theses and Dissertations (NDLTD) in Taiwan. The search strategy followed the PICO format, where P (patient or problem) included terms such as ‘care home’, ‘nursing home’, ‘residential aged care facility’, and long-term care facility’. I (intervention) comprised terms such as ‘Gold Standard Framework in Care Homes’, ‘integrated care pathway’, ‘care home project’, and ‘palliative care program’. The search period covered domestic and international literature published before 31 December 2023 (Table 1).

### 2.2. Eligibility Criteria

The present study’s inclusion criteria are studies focusing on interventions implemented in long-term care facilities (LCFs), utilizing approaches such as the Golden Standard Framework (GSF), Integrated Care Pathway (ICP), or other palliative care programs. Research designs may include both quantitative and qualitative methodologies. The exclusion criteria are studies involving populations from daycare centers or individuals not residing in institutionalized settings, studies presented in abstract format or inaccessible as full-text articles, and articles not published in English or Chinese.

### 2.3. Risk of Bias in Individual Studies

In this study, the quality of each research design was assessed using the Modified Jadad Scale. The Modified Jadad Scale consists of 8 items, each scored as either 1 or 0, resulting in a total score ranging from 0 to 8 points. The assessment criteria include whether randomization was described (1 point for yes, 0 points for no), whether randomization was appropriate (1 point for appropriate, 0 points for not described, −1 point for inappropriate), whether blinding was described (1 point for double-blinding, 0.5 points for single-blinding, 0 points for not described), whether blinding was appropriate (1 point for appropriate, 0 points for not described, −1 point for inappropriate), description of sample attrition rates and reasons, description of inclusion or exclusion criteria, description of adverse event assessment, and description of statistical analysis methods (1 point for described, 0 points for not described). A higher score indicates better study quality [9].

For non-randomized studies, we applied the Cochrane Risk of Bias Tool for Non-Randomized Studies of Interventions (ROBINS-I). This tool assesses biases related to confounding, the selection of participants, the classification of interventions, deviations from intended interventions, missing data, measurement of outcomes, and the selection of reported results. Each domain is evaluated for potential bias, which can impact the reliability of the study findings (Table 2) [1].

The qualitative studies included in our review were evaluated using the Standards for Reporting Qualitative Research (SRQR). This tool ensures that qualitative research is reported with clarity and comprehensiveness, covering aspects such as the research context, methodology, findings, and the interpretive validity of the research.

## 3. Results

### 3.1. Article Screening Results and Quality

A total of 38 articles were identified through a PubMed search, and 44 articles were found through an EMBASE search. After reviewing titles and abstracts, two duplicate articles and 24 articles unrelated to the topic were excluded. These included studies focusing on single populations or diseases, pain control, rehabilitation programs, cost analyses, and perspectives of healthcare workers. Additionally, 14 articles were excluded, as they did not meet the inclusion criteria, such as interventions conducted in hospital-integrated long-term care facilities or articles presented only as abstracts. Therefore, five articles were included for analysis.

**Figure 1:**
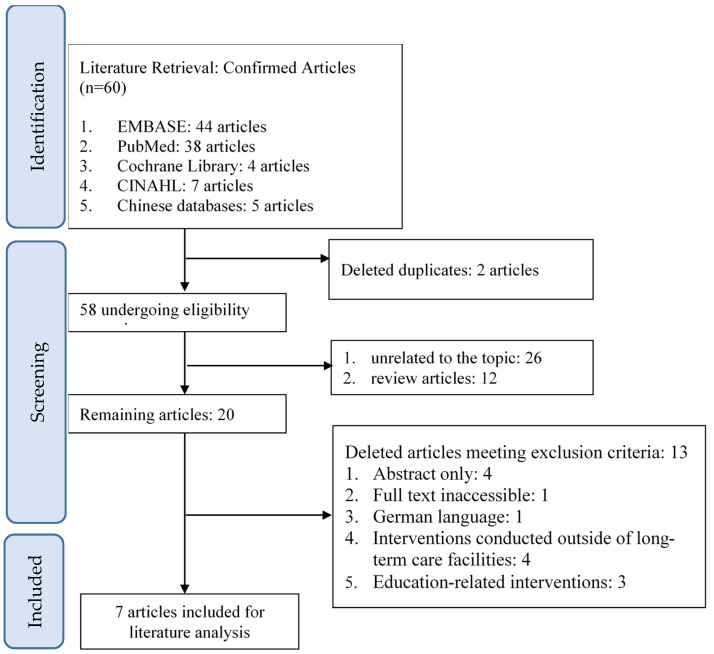
Literature screening process diagram.

Four articles were retrieved from the Cochrane Library, of which three were deemed irrelevant to the topic after reviewing titles and abstracts, such as studies focusing on discharge care system integration. Another article did not meet the inclusion criteria as it involved hospital and long-term care facility interventions. Hence, none of the Cochrane Library articles were included for analysis. No relevant randomized experimental literature was found in the Airiti Library or NDLTD in Taiwan. The literature search process is detailed in Figure 1.

Among the seven included articles, one was a controlled before-and-after study, four were cluster randomized controlled trials, one was a single-group pretest–post-test study, and one was a qualitative study. The evaluation results were organized and compared based on authors, publication years, study populations, study designs, interventions, outcome measures, study results, and literature quality. The quality assessment of the first four articles was conducted using the Modified Jadad Scale with scores ranging from 2 to 5 points. The remaining qualitative study article was evaluated based on the Standards for Reporting Qualitative Research (SRQR), meeting 13 out of 21 criteria (Table 3).

### 3.2. Study Design and Comparison of Study

#### Population and Interventions

Among the seven included studies, one was conducted in Sweden, two were European multinational comparisons, and the remaining studies were conducted in the United Kingdom. The publication years ranged from 2010 to 2021. There were six quantitative studies and one qualitative study. The total number of participating institutions was 188, involving 4671 participants, with an average age of 85.5 years. The institutional care staff comprised physicians, nurses, and care assistants, or only nurses and care assistants, all operating in 24 h residential care facilities.

In terms of interventions, two of the included studies primarily focused on the Program of All-Inclusive Care for the Elderly (PACE) [12]. This program was developed by the European Association for Palliative Care (EAPC) research group in 2015 with the registration number ISRCTN14741671 (FP7-HEALTH-2013-INNOVATION-1 603111). The PACE program consists of six steps, including discussions on end-of-life care preferences, assessment of care needs and review of care plans, integrated coordination of care plans, provision of high-quality care, end-of-life care, and bereavement support [12]. Further details can be found in Table 2. Additionally, two studies primarily focused on the Liverpool Care Pathway (LCP), while the remaining three explored the efficacy of the Golden Standard Framework in Care Homes (GSFCH), which inherently encompasses aspects of LCP. The results are organized according to themes evident in the text and Table 4.

Domains: D1: bias due to confounding, D2: bias due to selection of participants, D3: bias in classification of interventions, D4: bias due to deviations from intended interventions, D5: bias due to missing data, D6: bias in measurement of outcomes, and D7: bias in selection of the reported result. Judgement: moderate risk of bias, +: low risk of bias, ?: no information.

The duration of intervention implementation varied from 14 months to 3 years with the majority lasting for one year on average. Regarding the intervention content, the studies provided limited explanation regarding the standard content of LCP and GSFCH, as these involve official protocols and procedures. However, while GSFCH has standardized processes and content, its implementation primarily involves a one-year training program for institutions to achieve three objectives: enhancing the quality of palliative care for residents in long-term care facilities, strengthening the coordination of care among palliative care experts, institution staff, and community caregivers, and reducing unnecessary hospitalizations at end-of-life and increasing on-site palliative care [17]. However, in actual implementation, factors such as the actual visit frequency of professionals, cooperation of other professionals such as general practitioners (GPs) and district nurses (DNs), institutional cooperation, and operational stability can all affect the actual care outcomes. Among the included quantitative studies, only one study provided detailed explanations regarding these conditions, while the others did not provide much detail.

### 3.3. Outcome Indicators and Measurements

The measurement of outcome indicators included the assessment of various aspects such as quality of life (EQ-5D-5L), comfort (CAD-EOLD), symptom control effectiveness using the Edmonton Symptom Assessment System (ESAS), evaluation of services from informal caregivers using the Views of Informal Carers Evaluation of Services (VOICES) tool, quality of death (QOD-LTC), and cost-effectiveness analysis using net monetary benefit (NMB) [11,13,14,18]. Additionally, outcome measures included institutional end-of-life mortality rates, do-not-resuscitate (DNR) signing rates, completion rates of advance care planning (ACP), readmission rates, emergency admission rates, and staff effectiveness audits. Measurement time points for assessing outcomes were typically conducted at the conclusion of the intervention or within one month after the patient’s death except for baseline assessments. Evaluation methods primarily involved staff completing assessments, although questionnaires were also sent to close family members to gather perspectives from service recipients. Furthermore, one qualitative study utilized an action research methodology to investigate and understand the obstacles and difficulties long-term care facilities face in implementing the Liverpool Care Pathway (LCP) during the pre-, mid-, and post-implementation phases.

### 3.4. End-of-Life Outcomes of Palliative Care Programs

The results of the study indicated that the intervention of the PACE program did not yield statistically significant differences in quality of life between the two groups (group mean difference 2.7; *p* = 0.092). However, there was a significant difference in the quality of death outcomes (3.19 points, *p* = 0.00) [12]. In subgroup analyses, comparing dementia and non-dementia groups, no statistically significant differences were observed in comfort quality (mean subgroup difference 2.1; *p* = 0.177) and quality of death (−0.6; *p* = 0.741). Long-term care facilities (LTCFs) are gaining significance for end-of-life care in Europe, as extended durations of stay are associated with enhanced quality of care and comfort during the final month and week of life. Residents who have prolonged stays in LTCFs demonstrate higher probabilities of possessing advance directives and enduring power of attorney, underscoring the necessity for additional investigations aimed at comprehending and enhancing end-of-life care for all LTCF residents [19].

The implementation of the PACE program led to a significant reduction in costs by €983.28 (*p* = 0.020), which was primarily due to a decrease in hospital-related expenses (€919.51, *p* = 0.018) [10]. Regarding the implementation of the Liverpool Care Pathway (LCP), statistically significant differences were observed in symptom control for respiratory difficulty (−2.46; 95% CI = −4.43~−0.49) and nausea (−1.83; 95% CI = −3.12 ~−0.54). Additionally, there was a significant improvement in the degree of relief for respiratory difficulty (−0.47; 95%CI = −0.85 to −0.08). In community-based surveys featuring 3109 paired decedents, researchers found that patients treated by community-based specialist palliative care teams in the last 30 days of life had a mean health system cost that was $512 lower than those under usual care. Despite higher home care expenses, hospital costs were significantly reduced for the specialist team group. This suggests that community-based specialist teams can decrease health system costs, primarily due to lower hospital expenses, emphasizing the importance of palliative care assistance in a home setting [20]. However, many residents consider a nursing home to be their home.

In evaluating the effectiveness of the GSFCH program, several indicators showed improvement. (1) Death location: Study 4 indicated a decrease in in-hospital death rates from 15% to 8% before and after intervention, respectively, while Study 3 showed an increase in end-of-life rates within institutions from an average of 68% to 77.6%. (2) DNR signing rate: Study 4 showed an increase from 15% to 72% before and after intervention, respectively, while Study 3 showed an increase from an average of 32% to 59.3%. (3) LCP implementation rate: Study 4 showed an increase from 3% to 31% before and after intervention, respectively, while Study 3 showed an increase from an average of 4.6% to 29.3%. (4) ACP completion rate: Study 4 showed an increase from 4% to 53% before and after intervention, respectively, while Study 3 showed an increase from an average of 52.3% to 75.6%. (5) Readmission rate: The readmission rate in the last two weeks before death decreased from 31% to 24%. (6) Professional visitation: Family physicians accounted for the highest proportion of visits, comprising 96% of all visits [11,14,15].

The research identified six major barriers to implementing the LCP: (1) lack of relevant knowledge and skills in symptom control; (2) lack of readiness to deal with death; (3) inability to identify the timing of end of life and lack of understanding of the end-of-life process; (4) lack of multidisciplinary collaboration within institutions; (5) lack of confidence in discussing death; and (6) institutions are unprepared for change [16].

## 4. Discussion

This study employed a systematic literature review to explore the effectiveness of implementing palliative care interventions in long-term care facilities. A total of seven articles were included in the review. Literature analysis revealed that the implementation of palliative care interventions showed effectiveness in symptom control, end-of-life care quality, in-facility end-of-life rates, do-not-resuscitate (DNR) signing rates, advance care planning (ACP) completion rates, and inappropriate readmission rates. However, no differences were observed in comfort quality among the dementia care population. The measurement of outcome indicators should not only consider common symptoms and quality of comfort care but also take into account the unique characteristics of long-term care facilities, including diverse facility types, sizes, and care patterns [21,22]. Therefore, indicator measurement was tailored to the palliative care capabilities and resident characteristics of the facility, and different levels of indicators were established for evaluation to reflect the actual effects after intervention. Common difficulties encountered in implementing palliative care in long-term care facilities included “lack of palliative knowledge and interdisciplinary communication”, “insufficient resources for implementing palliative care”, “rational and inner conflicts”, and “facing decisions and reluctance regarding end-of-life care.” In addition to increasing palliative care training for facility staff, further consideration was given to how to reconstruct a stable service system for palliative care within the institutional framework. Utilizing existing home-based palliative care systems to provide services to residents meeting palliative care criteria within the facility fell short of meeting the needs of residents inside the facility and made it difficult to detect the needs of facility residents early on [23,24,25,26]. According to quality research, common challenges in implementing palliative care in long-term care facilities included “lack of palliative knowledge and interdisciplinary communication”, “insufficient resources for implementing palliative care”, “rational and inner conflicts”, and “facing decisions and reluctance regarding end-of-life care”. In addition to increasing palliative care training for facility staff, further consideration was given to how to reconstruct a stable service system for palliative care within the institutional framework [27]. Utilizing existing home-based palliative care systems to provide services to residents meeting palliative care criteria within the facility fell short of meeting the needs of residents inside the facility and made it difficult to detect the needs of facility residents early on.

Furthermore, due to the heavy workload and multitasking, home palliative care nurses find it challenging to conduct comprehensive assessments, screening, and palliative care services for institutions over the long term. According to the International Association for Hospice and Palliative Care (IAHPC), palliative care aims to improve the quality of life for patients, their families, and caregivers. Therefore, in terms of outcome indicators, the collection of patient-reported outcomes (PROs) is practically limited due to the changes in consciousness and limited mental and physical capacity of terminally ill patients. However, caregivers are also part of the care process. In a study defining good palliative care, caregivers stated that excellent end-of-life care services include providing nursing care and psychological support to patients; identifying and treating symptoms; ensuring continuity of care; respecting patients’ end-of-life wishes; providing environmental, emotional, and psychosocial support; keeping family members informed of the situation at all times; facilitating family understanding; and establishing partnerships with family caregivers through their involvement and guiding them in shared decision making. Therefore, in addition to strengthening the measurement of patient-reported outcomes, incorporating caregivers’ perspectives in future research is also an essential focus that should not be overlooked in research design.

## 5. Conclusions

Based on the findings of the studies reviewed, the implementation of palliative care interventions can enhance the quality of end-of-life care for residents. This includes the execution of the Liverpool Care Pathway (LCP), which effectively eliminates unnecessary medical interventions, reduces physical burden, and maintains bodily comfort and dignified end-of-life transitions. The Gold Standard Framework (GSF)-based GSFCH program assists institutional staff in early intervention by providing 6–18 months of education and collaboration, enabling the establishment of mechanisms and capabilities for caring for residents with terminal illnesses within institutions.

While the PACE program offers a comprehensive framework and serves as a reference for caregivers in designing interventions, its practical application requires a consideration of adjustments based on caregivers’ conditions, capabilities, and resources and existing institutional practices. Through collaborative efforts among team members, suitable and feasible implementation plans should be developed, which will be accompanied by specific, measurable indicators to reflect the effectiveness of the interventions.

In terms of practical care, government policy support plays a crucial role in the widespread implementation of such programs in the UK and Europe. Therefore, the implementation of these programs should also consider how to align with policy initiatives and establish relevant measures to ensure the sustainable integration of palliative care into institutional settings.

## Figures and Tables

**Table 1:** Search strategies.

Column Terms Combined With	Population AND	Intervention AND
OR	1 care home	6 Gold Standard Framework in Care Homes
OR	2 nursing home	7 integrated care pathway
OR	3 residential aged care facility	8 care home project
OR	4 long-term care facility	9 palliative care program
	5 combine 1–4 using ‘OR’	10 combine 6–9 using ‘OR’

Note: The final step combined steps 5 + 10 together using ‘AND’ to identify studies related to Palliative Care Interventions in Long-Term Care Facilities.

**Table 2:** Palliative care program in long-term care—PACE program.

Steps	Contents
Discussion of end-of-life care preferences	Discuss with residents and their families regarding end-of-life care preferences and choices, aiming to meet residents’ expectations.
2. Assessment of care needs and review of care plans	Conduct monthly discussions with physicians, nurses, and caregivers to review residents’ health status changes.
3. Integrated coordination of care plans	Organized multidisciplinary care meetings for residents with a life expectancy of less than six months to develop personalized plans addressing their physical, emotional, and spiritual needs and disseminate meeting outcomes to absent participants.
4. Provision of high-quality care	Provide training for caregivers on symptom management and communication skills, mainly focusing on pain and depression symptoms.
5. End-of-life care	Distribute end-of-life care guidelines to caregivers for reference during the terminal phase. These guidelines cover topics such as recognizing signs of impending death, communication with family and friends, psychological and spiritual support, and hydration issues.
6. Bereavement support care	Facilitate reflection and support meetings to provide caregivers with support and opportunities for experiential learning and exchange.

**Table 3:** Effectiveness of palliative care programs implemented in long-term care facilities.

Number	Author/Year/Country	Study Population	Study Design	Intervention Measures	Outcome Indicators and Measurement Tools	Research Findings	Literature Quality
1	Miranda et al., 2021 Belgium, United Kingdom, Finland, Italy, Netherlands, Poland, and Switzerland [10]	78 institutions/984 individuals (1) Control group: 558 individuals (2) Experimental group: 426 individuals (3) Average age at time of death: 85.9 years	Cluster RCT Experimental group: palliative care experts and institutional staff conducted a 2-month preparation, 6-month training, and 4-month consolidation for intervention	The PACE program trains institutional staff for a year in six steps, covering needs assessment, goal planning, symptom management, team meetings, and end-of-life care	Comfort Quality (CAD-EOLD) Quality of Death (QOD-LTC)	There was no significant difference in comfort quality between the dementia and non-dementia groups (mean difference: 2.1; *p* = 0.177) There was no significant difference in the quality of death between the dementia and non-dementia groups (mean difference: 2.7; *p* = 0.092)	5
2	Van Den Block et al., 2020 Belgium, UK, Finland, Italy, Netherlands, Poland, and Switzerland [11]	78 institutions/551 individuals (1) Control group: 272 individuals (2) Experimental group: 279 individuals (3) Average age at time of death: 85.45 years	Cluster RCT	The PACE program, lasting for one year, involves training institutional staff by palliative care experts through six steps, including assessing needs, setting goals, symptom management, team meetings, and end-of-life care	Quality of Life (EQ-5D-5L) Quality of Death (QOD-LTC) Cost-effectiveness analysis (net monetary benefit, NMB)	The two groups did not differ in terms of quality of life, but there was a significant increase in QOD-LTC outcomes (3.19 points, *p* = 0.00). The experimental group showed significant cost savings (€983.28, *p* = 0.020). The reduction in costs was primarily due to a decrease in hospital-related expenses (€919.51, *p* = 0.018)	5
3	Brännström et al., 2016 Sweden [12]	19 institutions/464 individuals (1) Control group: 220n = 71 (response rate: 55.9%) (2) Experimental group: 204n = 64 (response rate: 42.1%) (3) Average age: 86.1 years	Controlled before-and-after trial Improvement of end-of-life symptoms with LCP 15-month baseline follow-up Follow-up on family responses (from the family’s perspective) Description of patient and family characteristics	14-month LCP intervention	ESAS—Edmonton Symptom Assessment System VOICES—Views of Informal Carers—Evaluation of Services	Significant differences were observed in dyspnea (−2.46; 95% CI = −4.43 to −0.49) and nausea (−1.83; 95% CI = −3.12 to −0.54) The degree of improvement in dyspnea reached a significant difference (−0.47; 95% CI = −0.85 to −0.08)	low risk of bias
4	Kinley et al., 2014 United Kingdom [13]	38 institutions/divided into 3 groups (1) 12 institutions and managers participated in high-performance GSFCH and action learning (n = 804) (2) 12 institutions participated in high-performance GSFCH (n = 703) (3) observation group (n = 936) (4) Average age: 86.1 years	Cluster RCT Annual analysis of GSFCH effectiveness over 3 years	High facilitation and action learning GSFCH High facilitation GSFCH 3-year intervention period	In-hospital end-of-life rate LCP implementation rate DNR signing rate ACP completion rate	No significant difference in In-hospital end-of-life rate, LCP, DNR, ACP completion rate The intensity of intervention was significantly correlated with the achievement of GSFCH	5
5	Hockley et al., 2010 United Kingdom [14]	(1) 7 institutions (n = 228) (2) No grouping (3) Average age: 86.1 years	Single-group pretest–post-test Analysis of effectiveness indicators before and after the intervention	GSFCH (Gold Standard Framework in Care Homes) 18-month intervention period	In-hospital end-of-life mortality rate Hospital admission rate in the last two weeks of life DNR signing rate ACP completion rate LCP implementation rate	The in-hospital mortality rate decreased from 15% to 8% The readmission rate in the last two weeks of life decreased from 31% to 24% The DNR completion rate increased from 15% to 72% The ACP completion rate increased from 4% to 53%	low risk of bias
6	Kinley et al., 2014 United Kingdom [15]	(1) 38 institutions (2) n = 2444 (3) Average age: 85 years	Cluster RCT Analysis of the volume of professional services in the last 6 months	High facilitation and action learning GSFCH High facilitation GSFCH 3-year intervention period	Proportion of inappropriate admissions Number of visits by each professional Patterns of death	34% of residents were admitted to the hospital in the month before death, with 58% of these admissions being inappropriate GP visits accounted for 96%, while PCN visits accounted for 20% Patterns of death: ✓ 4.3% sudden death ✓ 50.3% decline ✓ 19.2% acute event ✓ 26.2% terminal 19% were readmitted within one month, 34% were readmitted within three months, and 56% died within one year	5
7	Watson et al., 2006 United Kingdom [16]	(1) 8 institutions (2) No grouping (3) Average age: not reported	Qualitative study—action research 5-year study on LCP, with an assessment of implementation barriers in the final year	Integrated care pathway	Not applicable	The analysis of barriers to implementing palliative long-term care interventions identified the following six factors: Lack of knowledge and skills in symptom control Insufficient preparation for encountering death Inability to recognize the timing of end of life and a lack of understanding of the dying process Lack of multidisciplinary teamwork within institutions Lack of confidence in discussing death Institutional unpreparedness for change	13/21

Abbreviations: ACP, advance care planning; Cluster RCT, cluster randomized controlled trial; DNR, do not resuscitate; GP, general practitioner; GSFCH, Golden Standard Framework Care Home; PACE, Program of All-Inclusive Care for the Elderly; PCN, palliative care nurse; LCP, Liverpool Care Pathway.

**Table 4:** Quality assessment for non-RCT (ROBINS-I tool).

First Author	Year	D1	D2	D3	D4	D5	D6	D7	Overall
Brännström	2015	-	+	-	?	-	+	+	+
Hockley	2010	-	+	-	+	+	+	+	+

## Data Availability

Data regarding the study are available upon request to the first author.
